# Surgical technique for preventing lung torsion after right upper and lower bilobectomy

**DOI:** 10.1093/icvts/ivad069

**Published:** 2023-05-09

**Authors:** Daisuke Eriguchi, Kentaro Imai, Naohiro Kajiwara, Norihiko Ikeda

**Affiliations:** Department of Thoracic Surgery, Hachioji Medical Center of Tokyo Medical University, Tokyo, Japan; Department of Thoracic Surgery, Hachioji Medical Center of Tokyo Medical University, Tokyo, Japan; Department of Thoracic Surgery, Hachioji Medical Center of Tokyo Medical University, Tokyo, Japan; Department of Surgery, Tokyo Medical University, Tokyo, Japan

**Keywords:** Postoperative lung torsion, Multiple primary lung cancer, Right upper and lower bilobectomy

## Abstract

In cases of right upper and lower bilobectomy, careful manipulation is required to avoid lung torsion, as only the right middle lobe remains in the right thoracic cavity. We report a case of successful right upper and lower bilobectomy with no torsion of the middle lobe. Our technique prevents postoperative lung torsion by fixing the lung to the chest wall and pericardial fat with silk threads. In situations where lung torsion is a concern after lung resection, fixing the remaining lungs with silk thread is effective in preventing lung torsion.

## INTRODUCTION

Lung torsion after lung resection is a rare but life-threatening complication. Especially, after the uncommon procedure of right upper and lower bilobectomy, lung torsion of the right middle lobe warrants attention. We present a novel, simple and successful technique for preventing lung torsion in cases of right upper and lower bilobectomy by a single-stage surgery.

## CASE PRESENTATION

**Figure 1:**
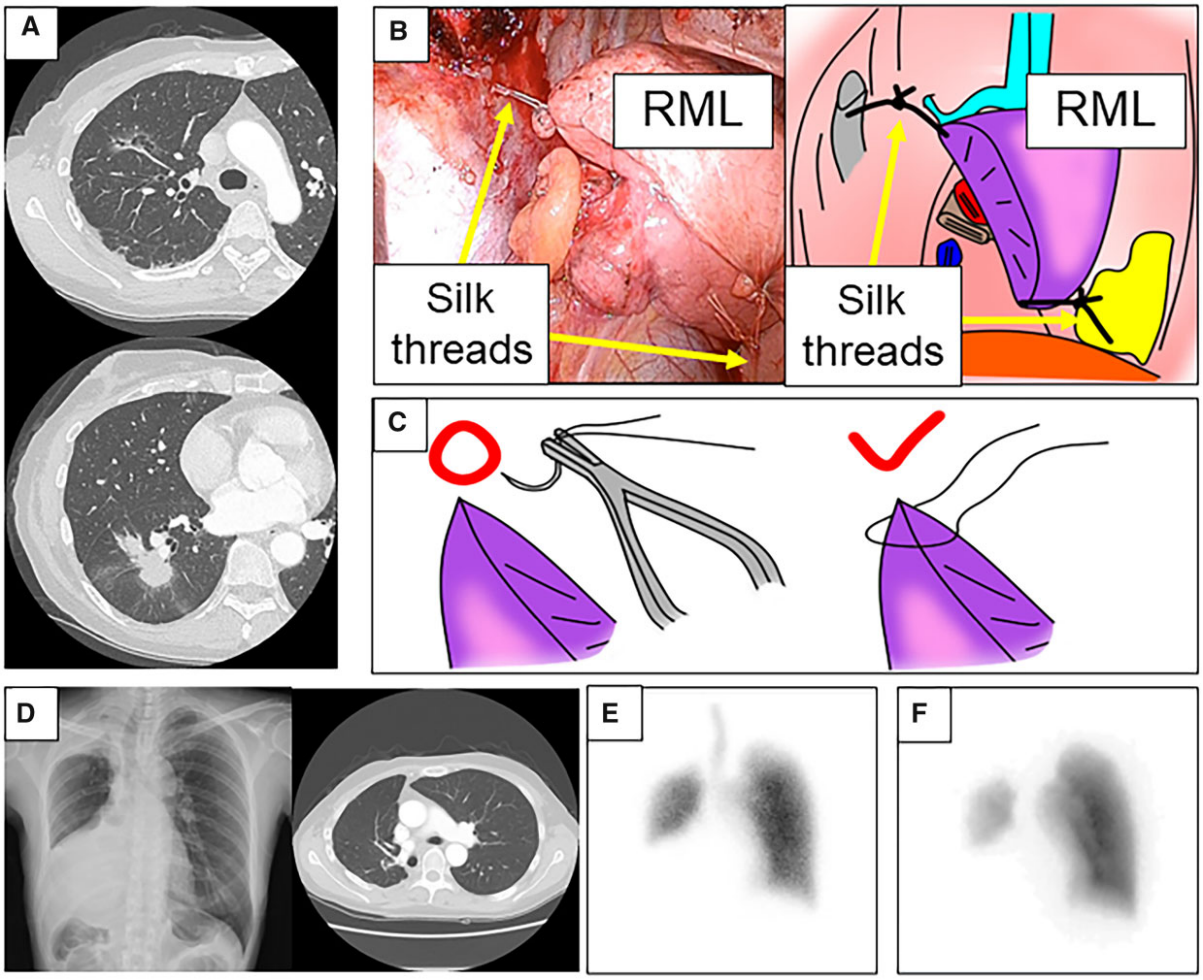
(**A**) Preoperative computed tomography. (**B**) Intraoperative finding and illustrations. Silk threads are ligated to connect the right middle lobe to pericardial fat and the chest wall to avoid torsion of the middle lobe. (**C**) In all fixations, ligations are performed across the visceral pleura rather than needle sutures to avoid pulmonary air leakage. (**D**) Postoperative chest radiograph and computed tomography at 6 months. (**E**) Lung ventilation scintigraphy. (**F**) Lung perfusion scintigraphy.

A 52-year-old woman was diagnosed with primary lung cancer. Chest computed tomography revealed 2 nodules in right S3 and right S6 areas (Fig. 1A), which we considered double primary lung cancer (upper nodule, c-T1bN0M0 stageIA2; lower nodule, c-T2aN0M0 stage IB). Preoperative forced vital capacity and forced expiratory volume in 1 s were 3250 and 2630 ml, respectively. Performing a complete resection to secure enough margins and avoiding lung cancer recurrence by sublobar resection were difficult. Thus, we performed a right upper and lower bilobectomy instead, along with right middle lobe fixation.

A posterolateral incision was made in the fifth intercostal space by cutting the sixth rib. There was no pleural adhesion. We performed right upper and lower bilobectomy and lymph node dissection. As the middle lobe was highly mobile, chances for lung torsion were greater. We used four 2–0 silk threads in 2 pairs to secure the middle lobe in 2 places in its original position. The thread centres of the first pair were ligated to the back of sixth rib, and the posterior part of the right horizontal fissure. For the second pair, the thread centres were ligated to the anterior part of the right oblique fissure and the pericardial fat near the diaphragm. Within each pair, the thread ends were connected so that the distance between the back of the sixth rib and the posterior part of the right horizontal fissure, and between the anterior part of the right oblique fissure and the pericardial fat near the diaphragm, would maintain the right middle lobe at its original position. To a certain extent, the middle lobe remained fixed at the original position without twisting (Fig. 1B and C). In all fixations, the lungs were ligated with silk threads by the entire visceral pleura, rather than sutured to the lung tissue with a needle. The operation lasted 182 min, with 20 ml of blood loss. The drain was removed on postoperative day 5 due to high drainage volume. Postoperatively, the patient recovered without complications and oxygen support.

The pathological findings confirmed the diagnosis of a double primary lung adenocarcinoma (upper nodule, T1aN0M0 stage IA1; lower nodule, T2aN2M0 stage IIIA). Postoperative chest radiography and computed tomography revealed the absence of torsion, good expansion and lung ventilation; postoperative perfusion scintigraphy showed normal perfusion in the right middle lobe (Fig. 1D–F). Postoperative forced vital capacity and forced expiratory volume in 1 s were 1890 and 1740 ml, respectively. After 6-month follow-up, the patient could receive postoperative adjuvant chemotherapy without problems and had a normal life.

## DISCUSSION

The incidence of lung torsion after lung resection is reportedly <0.4% [1]. Here, as there was only the right middle lobe in the right thoracic cavity after single-stage surgery, fixation of the right middle node was required. Even if a right S3 segmentectomy had been performed, the remaining upper and middle lobes would have been independent of each other, and there was concern regarding pulmonary torsion. In another case, a latissimus dorsi muscle flap was made and fixed to the thoracic cavity, and the right middle lobe was stabilized without free movement [2]. However, this method is difficult to perform and can lead to pulmonary infectious disease through large operative invasion. In our technique, lung torsion was easily prevented by silk threads that stabilized the right middle lobe to the mediastinum and chest wall, effectively preventing clockwise or counterclockwise lung torsion as it was fixed in 2 places. It is recommended that the entire pleura or the area where the stapler is applied be ligated and fixed by silk threads, as suturing the thread directly into the lung can cause air leak. The silk threads should be adjusted to a length not too tight, allowing the lungs slight mobility. Regardless, in cases of emphysema, our technique requires attention to air leaks. When our method did not succeed, we considered fixing the right middle lobe with artefacts or other means.

With complicated sublobar resection, we should be attentive to unexpected lung torsion. This method of 2 lung fixations by silk threads helps prevent lung torsion without compromising respiratory function.

**Conflict of interest:** none declared.

## Data Availability

The data underlying this article are available in the article and in its online supplementary material.
